# Establishment of Sandwich ELISA for Quality Control in Rotavirus Vaccine Production

**DOI:** 10.3390/vaccines10020243

**Published:** 2022-02-05

**Authors:** Cao Li, Guoxing Luo, Yuanjun Zeng, Feibo Song, Han Yang, Shiyin Zhang, Yingbin Wang, Tingdong Li, Shengxiang Ge, Ningshao Xia

**Affiliations:** 1 State Key Laboratory of Molecular Vaccinology and Molecular Diagnostics, School of Public Health, Xiamen University, Xiamen 361102, China; lililicao@stu.xmu.edu.cn (C.L.); luoguoxing@stu.xmu.edu.cn (G.L.); zengyuanjun@stu.xmu.edu.cn (Y.Z.); yanghan@stu.xmu.edu.cn (H.Y.); zhangshiyin@xmu.edu.cn (S.Z.); ybwang@xmu.edu.cn (Y.W.); nsxia@xmu.edu.cn (N.X.); 2 National Institute of Diagnostics and Vaccine Development in Infectious Diseases, School of Life Sciences, Xiamen University, Xiamen 361102, China; songfeibo@stu.xmu.edu.cn

**Keywords:** enzyme-linked immunosorbent assay, detection, P[4], P[6], P[8]-VP4*, vaccine

## Abstract

Non-replicating rotavirus vaccines are alternative strategies that may improve the protective efficacy of rotavirus vaccines in low- and middle-income countries. The truncated spike protein VP4 (aa26-476, VP4*)was a candidate antigen for the development of recombinant rotavirus vaccines, with higher immunogenicity and protective efficacy compared to VP8* and VP5* alone. This article describes the development of three genotype-specific sandwich ELISAs for P[4], P[6], and P[8]-VP4*, which are important for quality control in rotavirus vaccine production. Our results showed that the detection systems had good specificity for the different genotype VP4* and were not influenced by the *E. coli* host proteins. Moreover, the detection systems play an important role in determining whether the target protein was contaminated by VP4* proteins of other genotypes. They can also detect the adsorption rate of the adjuvant to the P[4], P[6], P[8]-VP4* protein during the process development. The three detection systems will play an important role in the quality control and process development of VP4* based rotavirus vaccines and facilitate the development of recombinant rotavirus vaccines.

## 1. Introduction

Rotavirus is the most common cause of severe diarrhea in infants and young children under 5 years [1], responsible for up to 200,000 child deaths each year [2]. Vaccination is the most efficient strategy for the control of rotavirus-associated diseases and mortality. Several live oral rotavirus vaccines have been approved by FDA and are widely used throughout the world [3]. Nevertheless, the protective efficacy of these rotavirus vaccines is decreased in low- and middle-income countries (LMICs), where rotavirus-related mortality is higher [4,5]. In recent years, studies have focused on non-replicating rotavirus vaccines (NRRV), which can be administered parenterally and may circumvent the reduced efficacy of live oral rotavirus vaccines [6,7].

A series of rotavirus antigens have been shown to be effective in animal models [8,9,10,11,12]. Among these antigens, the spike protein VP4, which mediates the attachment and penetration of rotavirus infection, was most widely explored [13]. VP4 can be digested into VP8* and VP5* in the presence of trypsin, and both VP8* and VP5* can stimulate neutralizing antibodies [12,14]. P[4], P[6], and P[8] are the most prevalent P genotypes of human rotaviruses [15,16,17,18], and a trivalent vaccine based on P2-VP8 is currently in clinical trials [7,19]. In a previous study, we proved that the VP4*stimulated higher titers of neutralizing antibodies and conferred higher protective efficacy than VP8* and VP5* alone, and VP4* could be a viable candidate for rotavirus vaccines [12].

Similar to P2-VP8, a trivalent formulation of VP4* should be developed to cover most of the prevalent rotavirus genotypes. Quantitative determination of the individual genotype VP4* proteins in the trivalent vaccine is important for both process development and quality control of the vaccine antigens. Monoclonal antibodies (mAbs) specific for VP4* were screened in our previous study [20], among which there were genotype-specific antibodies against P[4], P[6], and P[8]-VP4* proteins. In this study, we developed sandwich-enzyme-linked immunosorbent assays (ELISA) to detect different genotype VP4* antigens, which could be used for process development and quality control of VP4* antigens.

## 2. Materials and Methods

### 2.1. Expression and Purification of P[4], P[6], P[8]-VP4*

The nucleotide sequence of strains Wa (GenBank Accession # MG729832), MRC-DPRU3032 (GenBank Accession # KP752474), and 13-146 (GenBank Accession # MG729831) were selected as the coding sequence of truncated P[8], P[6], and P[4]-VP4 (aa26-476), respectively. The coding sequence of VP4* protein was synthesized and cloned into pTO-T7 plasmid by Sangon Biotechnology Co., Ltd. (Shanghai, China). For VP4* expression, the plasmid was transformed into *E. coli* Bl21(DE3) cells and expressed as previously described [21]. The cell pellets were resuspended in 50 mM Tris-HCl (pH8.0) and were lysed by sonication. After clarification, the VP4* proteins were purified from the supernatant by the following procedure. For P[4]-VP4*, 2 M CaCl_2_ was added to the supernatant to a final concentration of 40 mM, and 30 min later, the insoluble impurities were removed by centrifugation at 25,000× *g* for 10 min. Then, saturated ammonium sulfate was added to the supernatant to a final concentration of 40% and incubated on ice for at least 2 h. After centrifugation at 25,000× *g* for 10 min, the pellet was resolved by 50 mM Tris-HCl (pH 8.8), and the soluble fraction was further purified by a two-step high-performance Q and phenyl 6 FF (GE, Sweden) chromatography as described in previous study [12]. For P[6]-VP4* and P[8]-VP4*, the target proteins were purified using Q Sepharose High Performance and phenyl 6FF columns as previously described [12]. The purity of the proteins was assessed by SDS-PAGE (Supplementary Figure S1), and the concentration was measured by a BCA assay (Thermo Fisher Scientific, Rockford, IL, USA) according to the manufacturer’s instructions.

### 2.2. Monoclonal Antibody Generation and Production

Monoclonal antibodies (mAbs) specific for VP4* proteins were screened in our previous studies [20]. Briefly, 6-week-old BALB/c mice were immunized with the mixture of 60 μg P[4]-VP4*, P[6]-VP4* and P[8]-VP4* formulated with aluminum adjuvant (the ratio of P[4]:P[6]:P[8]-VP4* protein was 1:1:1). The mAbs were screened by enzyme-linked immunosorbent assay (ELISA) and micro-neutralization assay (ELISPOT). After three rounds of cloning, the stable clones were inoculated to mice, and the mAbs were purified from the ascites using protein A [22]. The mAb screening and preparation protocol was approved by Xiamen University Laboratory Animal Center. The binding reactivity of these mAbs to the three genotype VP4* proteins was determined by ELISA. A total of 50 mAbs were screened, and the genotype-specific highly reactive mAbs (Supplementary Table S1) were selected in this study for establishment of the genotype-specific detection system.

### 2.3. Enzyme-Linked Immunosorbent Assay

Sandwich ELISA was established for detection of specific genotype VP4* proteins. Briefly, the best suitable coating antibody and detection antibody were determined by checkerboard. The 96-well microplates were coated with 100 μL of 4 μg/mL antibody diluted in 20 mM phosphate buffer (pH7.4) at 37 °C for 2 h. After blocking, 100 μL of P[4], P[6], or P[8]-VP4* diluted in 20% newborn bovine serum (NBS) diluted in 50 mM Tris-HCl (pH 8.8) were added to each well and incubated at 37°C for 30 min. Unadsorbed VP4* proteins were removed by washing five times with PBST (0.05% Tween 20 in PBS). Then, 100 μL of 1 μg/mL detection antibody, which was labeled with horseradish peroxidase (HRP) by sodium periodate oxidation method [23], was added. After incubation at 37 ℃ for 30 min, unbound detection antibodies were removed by washing five times with PBST. Finally, these wells were incubated with 100 μL per well of TMB substrate solution in dark at 37 ℃ for 15 min. Fifty microliters per well of 2 M H_2_SO_4_ was added to terminate the reaction. Optical density (OD) at 450 nm with 630 nm as reference was determined using microplate reader (TEACAN, Männedorf, Switzerland).

For quantitative detection, a standard curve was included in each microplate. Briefly, the P[4], P[6], or P[8]-VP4* proteins were serially diluted by 1.5-fold dilutions in 20% NBS, three repeats for each concentration. All data were log_10_ transformed, and the standard curves were plotted by linear regression (GraphPad Software, Inc., La Jolla, CA, USA). The concentration of each sample was calculated from the corresponding standard curves.

### 2.4. Vaccine Adsorption Experiment

Aluminum adjuvant was kindly presented by Innovax (Xiamen, China). Na_2_HPO_4_, NaCl, double distilled water, and VP4* proteins were added to aluminum adjuvant in sequence to prepare the final formulation with 1 mg/mL Al, 2.35 mmol/L P, 150 mmol/L NaCl, and 60 or 120 μg/mL VP4* proteins. After mixing, the formulation was stood at 2–8 °C for at least 4 h for adsorption. Then, the mixture was centrifuged at 2000× *g* for 10 min, and the supernatant was taken for ELISA detection. All samples were detected in duplicate, and the adsorption rate was calculated according to the formula: (1-mean concentration in the supernatant/total concentration) × 100%.

## 3. Results

### 3.1. The Establishment of Double-Antibody Sandwich ELISA

**Figure 1:**
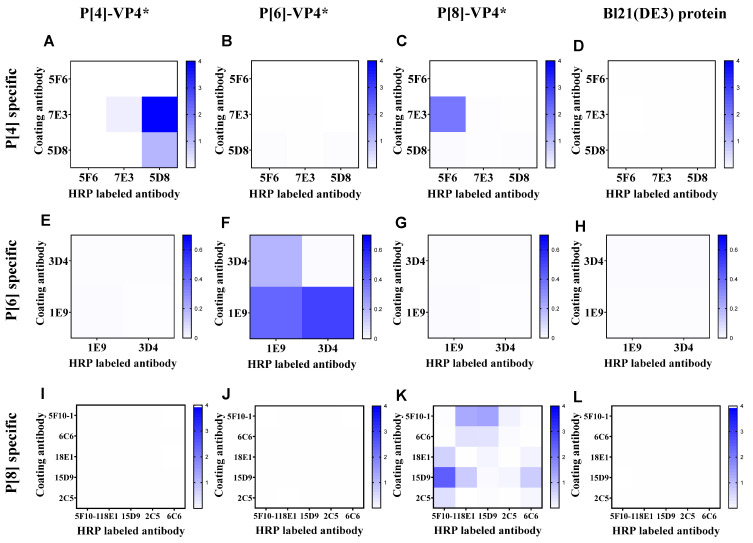
The reactivity of the antibody pairs for P[4], P[6], P[8]-VP4*, and Bl21(DE3) *E. coli* lysate as examined by checkerboard. (**A**–**D**) The reactivity of P[4]-VP4*-specific mAb pairs for detecting 100 ng/mL P[4]-VP4*, 100 ng/mL P[6]-VP4*, 100 ng/mL P[8]-VP4*, and the cell lysate of Bl21(DE3); (**E**–**H**) the reactivity of P[6]-VP4*-specific mAb pairs for detecting 50 ng/mL P[4]-VP4*, 50 ng/mL P[6]-VP4*, 50 ng/mL P[8]-VP4*, and the cell lysate of Bl21(DE3); (**I**–**L**) the reactivity of P[8]-VP4*-specific mAb pairs for detecting 20 ng/mL P[4]-VP4*, 20 ng/mL P[6]-VP4*, 20 ng/mL P[8]-VP4*, and the cell lysate of Bl21(DE3).

According to the binding activity of the mAbs, 4, 2, and 6 mAbs specifically reacted with P[4]-VP4*, P[6]-VP4*, and P[8]-VP4* were selected to establish sandwich ELISA for the detection of genotype-specific VP4* (Supplementary Table S1). All the mAbs were labeled with HRP, and the best mAb pair for each genotype of VP4* was determined by the checkerboard method. The antibody pair 7E3: 5D8-HRP showed the highest reactivity to P[4]-VP4* (Figure 1A) and no cross-reactivity to P[6]-VP4*, P[8]-VP4* or the *E. coli* host proteins (Figure 1B–D). Thus, 7E3: 5D8-HRP was selected for the detection of P[4]-VP4*. Similarly, 1E9: 3D4-HRP and 15D9: 5F10-HRP were selected for detection of P[6]-VP4* and P[8]-VP4*, respectively (Figure 1E–L).

**Figure 2:**
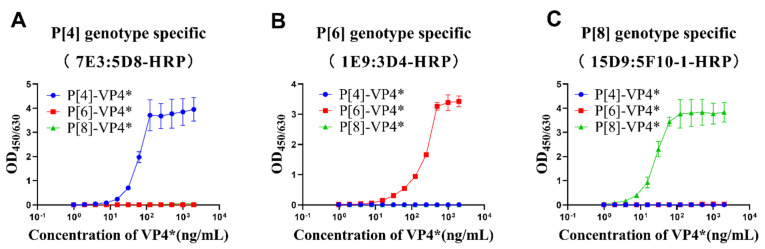
The detection range and specificity of P[4], P[6], and P[8]-VP4*-specific detection systems. The purified VP4* proteins were 2-fold serially diluted from 2 μg/mL to 0.97 ng/mL by 20% NBS, and detected by P[4], P[6], and P[8]-specific VP4* detection system. (**A**) The reactivity of P[4]-VP4* antibody pair 7E3:5D8-HRP to different genotype VP4* proteins; (**B**) the reactivity of P[6]-VP4 antibody pair 1E9:3D4-HRP to different genotype VP4 proteins; (**C**) the reactivity of P[8]-VP4* antibody pair 15D9:5F10-1-HRP to different genotype VP4* proteins. All the experiments were performed in three replicates, and the error bars represent the standard deviation of each group.

P[4]-VP4*, P[6]-VP4*, and P[8]-VP4* were two-fold serially diluted and detected by the selected antibody pairs to determine the linear range and cross-reactivity of the three antibody pairs. The results show that OD values increased with the increases in the corresponding genotype VP4* proteins, while no cross-reactivity with the other two genotypes, even at the highest concentration tested, was observed (Figure 2). The P[8]-VP4* detection was most sensitive, with a linear range of 2–30 ng/mL, followed by P[4]-VP4* (8–60 ng/mL) and P[6]-VP4* (15–250 ng/mL) (Figure 2). The potential applications of the sandwich ELISAs were further evaluated.

### 3.2. Detection of VP4* in E. coli Lysate by the Sandwich ELISAs

**Figure 3:**
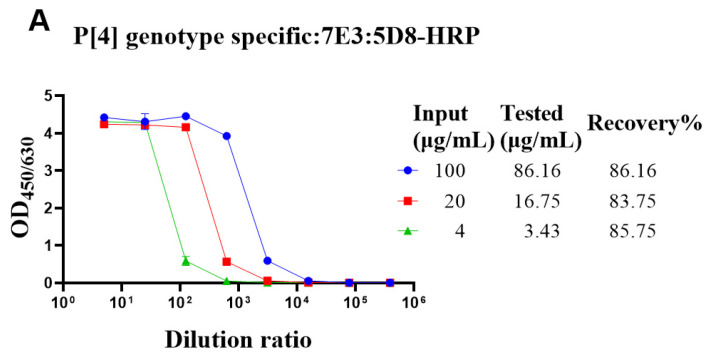

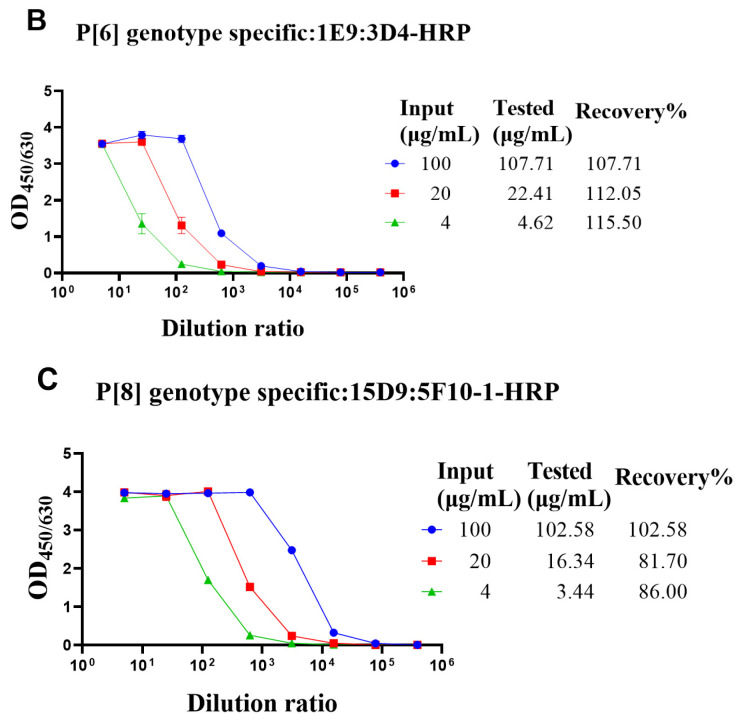
The recovery rate of P[4], P[6], and P[8]-VP4* proteins in *E. coli* lysate. The purified P[4], P[6], and P[8]-VP4* proteins were added to the supernatant of Bl21(DE3) *E. coli* lysate to a final concentration of 100, 20, and 4 μg/mL, respectively, and detected by the corresponding genotype-specific antibody pairs. (**A**) The results detected by P[4]-VP4*-specific mAb pair (7E3: 5D8-HRP); (**B**) the results detected by the P[6]-VP4*-specific mAb pair (1E9: 3D4-HRP); (**C**) the results detected by the P[8]-VP4*-specific mAb pair (15D9: 5F10-1-HRP). All the experiments were performed in three replicates, and the error bars represent the standard deviation of each group. The concentration of each protein was calculated from the corresponding standard curve (Supplementary Figure S2). The recovery rate was calculated according to the formula: VP4* _calculated_/VP4*_theoretical_*100%.

The genotype-specific detection system could be useful in optimizing the fermentation conditions of VP4* proteins. Though there was no cross-reaction with the bacterial proteins, it was still not sure whether bacterial proteins could interfere with the detection. To determine whether the bacterial proteins could interfere with the detection of VP4* proteins, the lysate of Bl21 (DE3) was spiked with different concentrations of VP4* proteins, and after dilution, the spiked samples were detected by each antibody pair for VP4*. The results show that the recovery rate was between 80% and 120% (Figure 3), indicating that the interference of bacterial proteins could be neglected, and the assay could be used for optimizing the fermentation conditions and purification processes. 

### 3.3. Identity Evaluation of Purified VP4* Proteins Using the Sandwich ELISAs

**Figure 4:**
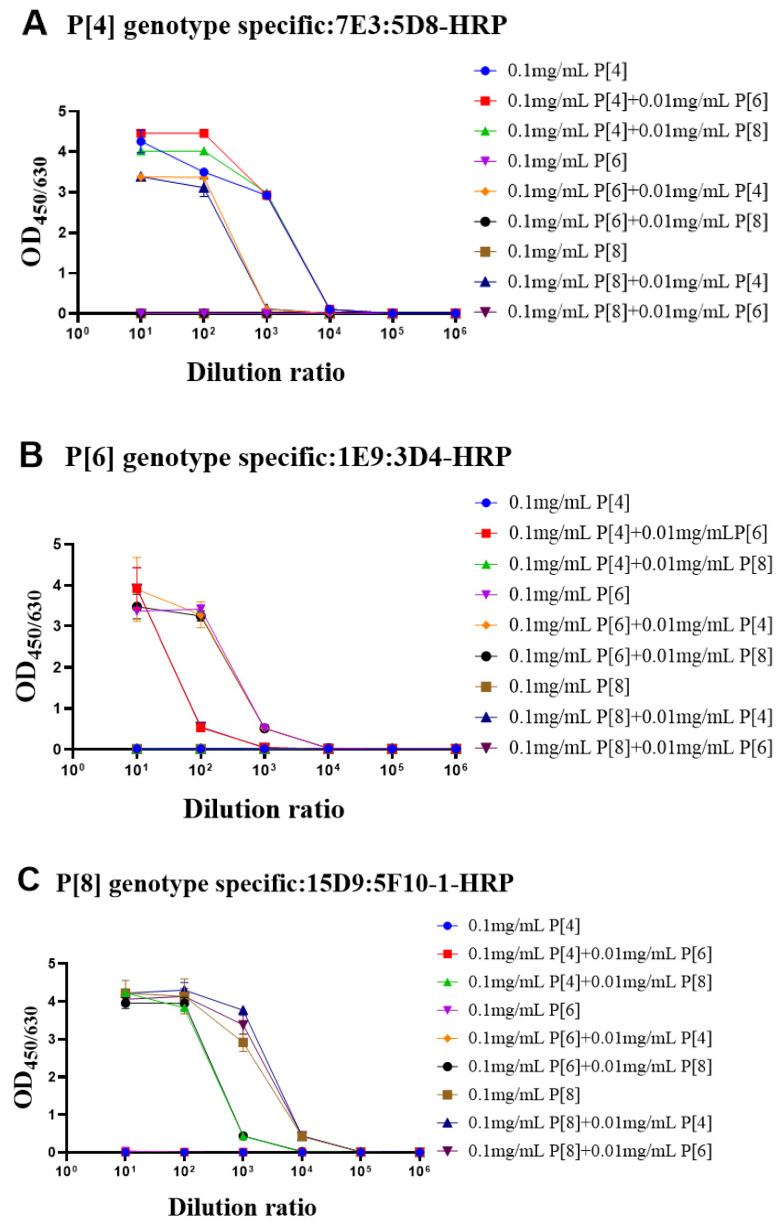
The evaluation of the identity of purified VP4* proteins by the genotype-specific mAb pairs. In order to confirm whether the ELISA systems can be used to detect protein contamination, we mixed 10 μg/mL of the other two VP4* proteins into P[4], P[6], and P[8]-VP4* protein, respectively, and then the mixtures were detected by the three genotype-specific mAb pairs. As shown in the (**A**–**C**), the three ELISA detection systems can accurately identify the P[4], P[6], and P[8]-VP4* in the mixed samples. All the experiments were performed in two replicates, and the error bars represent the standard deviation of each group.

During the fermentation or purification process, the proteins could be contaminated by other bacteria or proteins. Thus, it was important to determine whether the target protein was contaminated by VP4* proteins of other genotypes. To simulate contamination, the purified target genotype VP4 proteins were mixed with other genotype VP4* proteins (100 μg/mL: 10 μg/mL). The single-genotype VP4* proteins and protein mixtures were detected by each antibody pair after 10-fold serial dilution. As shown in Figure 4A, when detected by the P[4]-VP4*-specific antibody pairs, there was no significant difference in the detection signals of the P[4]-VP4* protein after the addition of P[6]-VP4* or P[8]-VP4*. The additionally added P[6]-VP4* and P[8]-VP4* proteins could be detected in the P[6]-VP4* and P[8]-VP4*-specific detection system (Figure 4B,C). Thus, if the target protein was contaminated by other genotypes, it could be identified by the genotype-specific sandwich ELISA. 

### 3.4. Determining the Adsorption Rate of VP4* Proteins on Aluminum Adjuvant

It was also important to determine the adsorption rate of each protein during the process development. To evaluate the adsorption rate of each genotype’s VP4* proteins on aluminum adjuvant, the formulations containing VP4* proteins and aluminum adjuvants were centrifuged, and the unadsorbed VP4* proteins were detected by the genotype-specific sandwich assays. As shown in Table 1, the adsorption rate for each genotype VP4* was above 98% at all the conditions tested.

## 4. Discussion

Genotype-specific quantitative detection of VP4* proteins is important for process development and the quality control of recombinant VP4*-based trivalent rotavirus vaccines. In this study, three sandwich ELISAs for quantitative detection of genotype-specific VP4* proteins were developed based on genotype-specific mAbs.

ELISA was commonly used in the characterization and quality control of vaccines, including antigenicity analysis, immunogenicity evaluation, structural qualification, etc. [24,25,26,27]. For multivalent vaccines, it was crucial to determine the content of each antigen in the formulation. Though total protein concentration could be determined by many methods, such as UV absorbance, Bradford assay, and BCA assay, it was unable to determine the concentration of each antigen. Based on type-specific mAbs, it was feasible to detect specific antigens in the vaccine formulation. Quantitative ELISAs were developed for Neisseria meningitidis vaccines and tetanus vaccines in previous studies [28,29,30]. 

In this study, the sandwich ELISAs were used to determine the adsorption rate of VP4* antigens in the aluminum adjuvant formulation, which were simple and highly specific. They can specifically determine the adsorption rate of each genotype’s VP4* antigen on aluminum adjuvant. In the trivalent vaccine mixture, the adsorption rate of each genotype’s VP4* proteins could be determined separately. David McAdams et al. [24] also established three inhibition ELISAs for vaccine adsorption rate detection. The sandwich ELISAs in this article had higher sensitivity compared with the inhibition ELISAs. In addition to determining the VP4* antigen adsorption rate, the assays we established will be useful for monitoring the lot-to-lot consistency and stability of different VP4* antigens in vaccine stocks.

The quantitative ELISAs could also be used for process development and quality control of intermediate products. Though SDS-PAGE and Western blot could also be used to identify the expression of a target protein, it was time-consuming and difficult to determine the accurate expression level, while ELISA was simpler and more accurate for quantitation. As shown in Figure 3, the recovery rate of VP4* in *E. coli* lysate was between 0.8 and 1.2, and the impact of bacterial proteins on the detection of VP4* could be neglected.

In summary, three genotype-specific quantitative ELISAs were developed for the detection of P[4], P[6], and P[8]-VP4* proteins, which were proven to be viable candidates for NRRV vaccines and may confer higher protective efficacy in LMICs. Our results showed that the quantitative assays could be used for determining the adsorption rate of each protein and monitoring the identity of the vaccine stocks. The assays could be used for process development and quality control of trivalent VP4* vaccines, which will facilitate the development of VP4*-based rotavirus vaccines.

## Figures and Tables

**Table 1:** The adsorption rate of VP4* antigens *.

Vaccine Composition Total VP4* (μg/mL)	P[4]-VP4*		P[6]-VP4*		P[8]-VP4*		P[4]+P[6]+P[8]-VP4*	
	60	120	60	120	60	120	180	360
Unadsorbed P[4]-VP4* (μg/mL)	0.32	0.59	/	/	/	/	0.40	1.50
Unadsorbed P[6]-VP4* (μg/mL)	/	/	0.39	1.52	/	/	0.80	2.00
Unadsorbed P[8]-VP4* (μg/mL)	/	/	/	/	0.22	0.92	0.23	0.97
Adsorption rate (%)	99.47	99.51	99.35	98.73	99.63	99.23	99.21	98.76

*: The adsorption rate was calculated as (1-adsorbed VP4*/Total VP4*) × 100%, and the results were expressed as the mean of the two replicates of each experiment.

## Data Availability

Not applicable.

## Supplementary Materials

The following supporting information can be downloaded at: www.mdpi.com/article/10.3390/vaccines10020243/s1, Table S1. Reactivity of monoclonal antibodies (mAbs) to the three genotype recombinant VP4* antigens. Figure S1. SDS-PAGE characterization of purified P[4], P[6], P[8]-VP4*. Figure S2. Standard curve of sandwich ELISA for P[4], P[6], P[8]-VP4*.
