# Supplementary material for: Establishment of Sandwich ELISA for Quality Control in Rotavirus Vaccine Production
Source: Vaccines (Basel). 2022 Feb 5;10(2):243. doi: 10.3390/vaccines10020243 (PMC8876306; doi:10.3390/vaccines10020243)
Supplement: Supplementary file 1 [file vaccines-10-00243-s001.zip › Supplementary figures-R1.pdf]

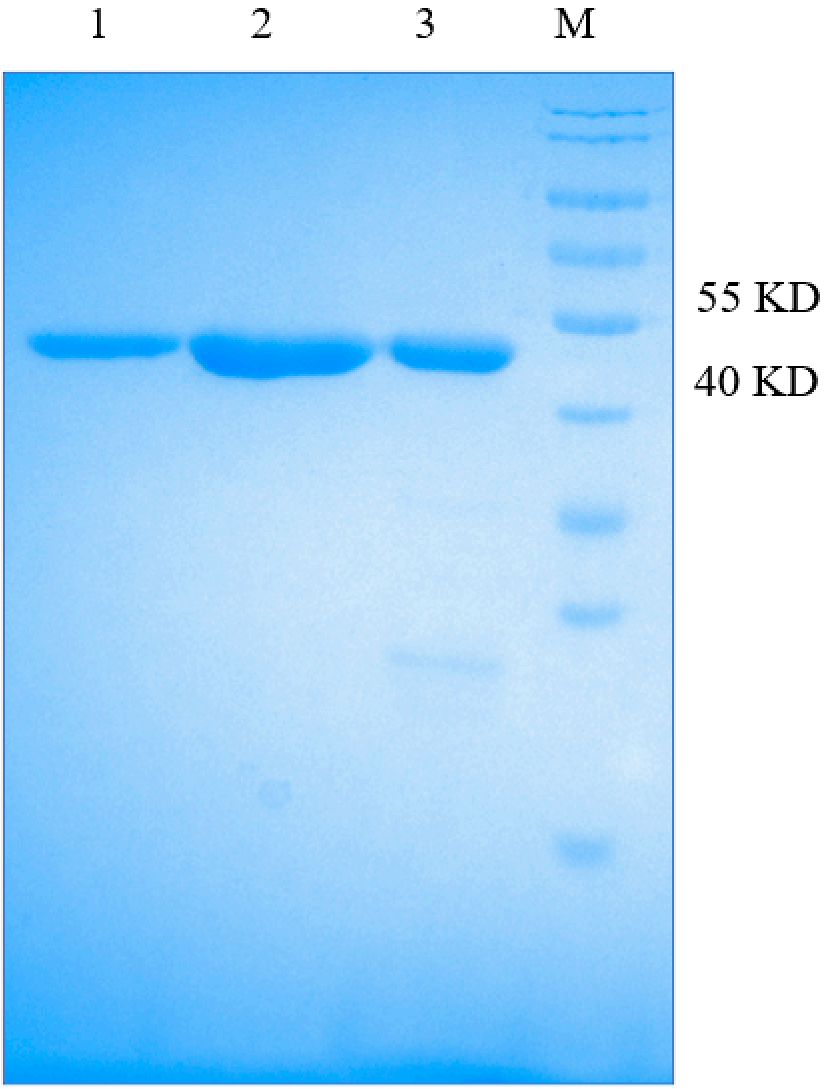

Figure S1. SDS-PAGE characterization of purified P[4], P[6], P[8]-VP4*. Lanes 1-3 represent the samples of purified P[6]-VP4*, P[8]-VP4*, P[4]-VP4* proteins respectively.

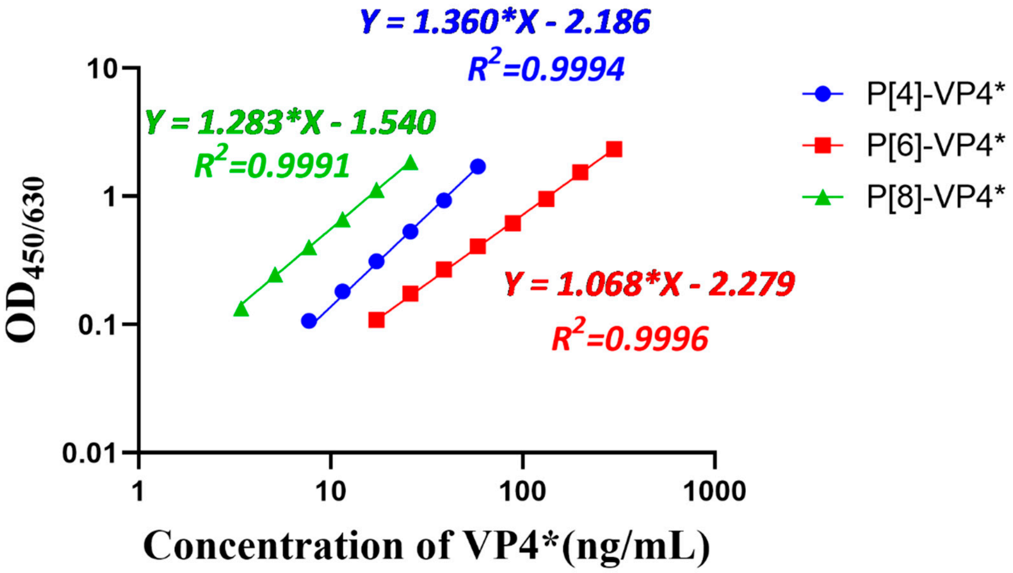

Figure S2. Standard curve of sandwich ELISA for P[4], P[6], P[8]-VP4*. Purified VP4* proteins were 1.5-fold serially diluted. The error bars represent the standard error of each group (n=3).
