# Supplementary material for: Establishment of Sandwich ELISA for Quality Control in Rotavirus Vaccine Production
Source: Vaccines (Basel). 2022 Feb 5;10(2):243. doi: 10.3390/vaccines10020243 (PMC8876306; doi:10.3390/vaccines10020243)
Supplement: Supplementary file 1 [file vaccines-10-00243-s001.zip › Table S1.pdf]

Table S1. Reactivity of monoclonal antibodies (mAbs) to the three genotype recombinant VP4* antigens

Clone	Subclass	Means	EC_{50} (µg/mL) *		
			P[4]-VP4 *	P[6]-VP4 *	P[8]-VP4 *
7E3	IgG2a	ELISA	1.124	>100	97.01
5F6	IgG2b	ELISA	1.29	23.18	21.27
5D8	IgG1	ELISA	0.01	>100	>100
1E9	IgG1	ELISA	>100	0.04	>100
3D4	IgG1	ELISA	>100	0.27	>100
18E1	IgG2b	ELISA	22.21	15.8	0.52
2C5	IgG1	ELISA	35.26	87.32	0.31
6C6	IgG2b	ELISA	>100	>100	0.47
15D9	IgG2b	ELISA	31.62	26.3	1.32
5F10-1	IgG1	ELISA	33.2	39.62	0.18

* The binding activity of mAbs to VP4 protein belonging to different P genotype. The EC_{50} value for each mAb combination was shown. Sample with no reactivity are indicated by ">100" (the highest tested mAb concentration).
