# Supplementary figures and images for: Establishment of Sandwich ELISA for Quality Control in Rotavirus Vaccine Production
Source: Vaccines (Basel). 2022 Feb 5;10(2):243. doi: 10.3390/vaccines10020243 (PMC8876306; doi:10.3390/vaccines10020243)

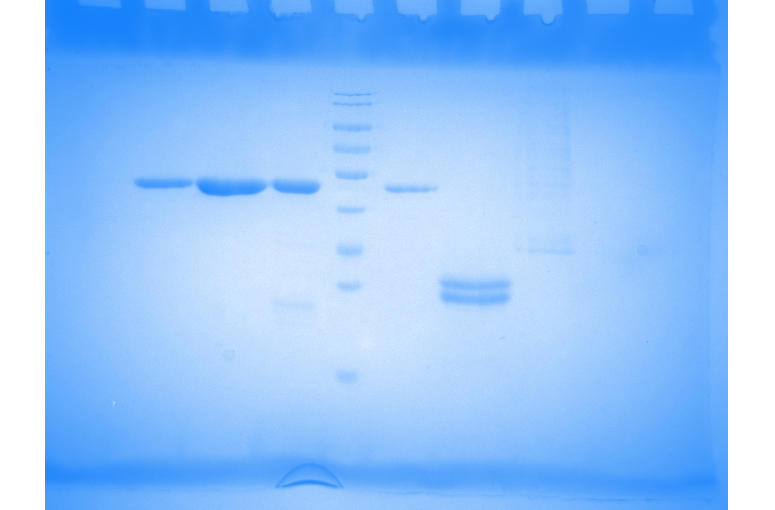

Supplement: Supplementary file 1 [file vaccines-10-00243-s001.zip › The original Figure S1.tif]
